# Unpacking emotional contexts of post-truth.

**DOI:** 10.1080/19460171.2019.1670222

**Published:** 2019-09-25

**Authors:** Anna Durnová

**Affiliations:** Department of Public and Social Policy, Charles University & Institute for Advanced Studies, Vienna, Austria

**Keywords:** Discourse, emotions, post-truth, science denial, social constructivism

## Introduction

We have landed in times when being in a rage against expertise is repeatedly highlighted as a legitimate political stance. While expertise has become an inherent part of policy-making, the public discourse around expertise has raised waves of criticism because of the alleged ideological biases of expertise, opportunistic strategies or contradictions in what evidence should be followed by governments and policy actors. These debates are not new. They recall some of the vivid battles between positivist and interpretive traditions around the production of knowledge in policy studies (see the discussion in this journal in: Jones and Radaelli 2015; Dodge 2015). They also bring back on the agenda the debate around relativizing tendencies of social constructivists, who supposedly cause damage to the scientific search of ‘truth’ (Shore 2017).

Nonetheless, these debates now seem to be highly pending . The scholarship around critical policy studies has been at the forefront in highlighting how characterizing social constructivism through relativism is a substantial misperception of this epistemological orientation in social science. Critical policy studies want to pay closer attention to the ways in which expertise is embedded in social relations (Strassheim 2017). Social embeddedness encompasses power relations on that expertise reacts, and that it seeks to revise or further consolidate (Fischer 2009). While being the main tool of policymakers, ‘evidence’ has a contradictory path that has been analyzed through critical inquiry.

One of these contradictions is carefully analyzed in Frank Fischer’s contribution to the discussion around science denial as one of the main phenomenon related to the alleged post-truth era (Fischer 2019). While post-truth is being presented in public discourse around science denial as a threat to science and to its principles, Fischer argues that observing the debate on facts from the perspective of social constructivism allows us to reveal deeper socio-political reactions and interpretations of facts. The observation from the perspective of social constructivism explains that its epistemological orientation to knowledge-making is not the culprit of a post-truth world but a lens that enambles scholars to better understand where the denial of facts comes from. It can thus enlighten political implications of such a denial because it shows the role of discourse, culture and social context in the legitimacy of expertise (Jasanoff and Simmet 2017; Nowotny 2015).

The importance of such analysis of socio-cultural aspects of science denial when understanding the post-truth phenomenon can be further understood and elaborated upon by highlighting the role of ‘discourse on emotions’ (Durnová 2019b). How emotions are portrayed and represented in the discourse on science often separates its actors into those who ‘know the facts’ and those who are merely ‘emotional’. That emotions and fact making are interrelated and complementary has been extensively explained elsewhere (Ahmed 2013; Zerilli 2012). I want to highlight here the public manifestation of a division between emotions and facts that has serious implication for the societal polarization occurred as a by-product of post-truth.

Emotions have recently gained prominence in social sciences (see overviews of these discussions in: Czarniawska 2015; Jasper 2011; Wouters 2012). Central to such arguments is that emotions are endowed with meanings that we uncover through analyzing how emotions are referenced in public discourse and how, through these specific references, they frame what is being said as ‘rational’, ‘irrational’, ‘factual’ or ‘emotional’ (Durnová 2018). In the particular case of science denial, such referencing is part of qualifying knowledge as ‘fact’ as opposed to ‘emotion’ or ‘mere opinion’.

## Binary opposition of facts and emotions

Opposing ‘facts’ to ‘emotions’ without further specification of which emotions are meant – as done in the characterizations of post-truth (Fish 2016; Higgins 2016) – has engendered a binary opposition of facts and emotions in the discourse on post-truth (Durnová 2019b). Discourse on post-truth is closely related to a discourse on a societal division of a fact-oriented elite represented mainly by ‘experts’ that are in the discourse on science opposed to the ‘emotional’ or even ‘ignorant’ public. The use of the term ‘public’ often conflates those who deny the science or those who fear it. Binary opposition produces a public discourse, in which facts are not emotions and emotions are not facts. In such way, the binary opposition becomes a powerful discursive strategy influencing the legitimacy of actors taking part in the public debate on science. You can not be portrayed as emotional, if you want to be placed on the same side as facts.

As we see in Fischer’s analysis, social constructivism enables us to see that both sides of the public discourse on science denial have an emotional response and both use it in their messages. Both sides also spin the message as pointed out in the example of the hacked emails of climate scientists between 2009 and 2011, which has among others showed that scientists do debate how to frame their evidence so that the message gets across (Fischer 2019). Yet, what is important here is the way in which the discourse on emotions is used to de-legitimize opposition. The science denial discourse uses such discourse on emotions to portray experts ‘as part of conspiracy’ as a ‘liberal urban intelligentsia’ (examples mentioned in Fischer’s contribution), which are part of loaded language that should highlight bad intentions and arrogance. Such discourse creates an image of experts that use science as a part of opportunistic strategy that helps them gain a legitimate position in public discourse. The same strategy dominates scientists’ critique of the Trump presidency. Its important part being that Trump is criticized not for ideology affiliation, but for his dislike of facts, for his repeated attacks on science and for being ‘irrational’, ‘emotional’. While emotions on the side of scientists and those who support them in the critique of Trump are connoted positively in the discourse on science – as the necessary component to get the message across – the emotions of the public, mainly fear and anxiety when related to the facts around climate change, are framed as ‘irrational’ or ‘ignorant’ behavior.

Putting emphasis on the discourse on emotions in the analysis of post-truth is complementary to the analysis of socio-cultural aspects of science denial and post-truth more generally. Emotions are connected to particular social and cultural representations, and the discourse on emotions use these to frame actions as ‘emotional’. Similarly, actors taking part in the public are not separable from their social status and cultural identity, and these components are transmitted by specific references to emotions. We find these components in other discussions around post-truth. The June 2016 Brexit vote was repeatedly cited as an illustration of the dichotomy between ‘the emotional people’ versus ‘experts’ (Polletta and Callahan 2017; Hochschild 2016). The underlying argument of that dichotomy was that knowledge was placed in the hands of experts who should, or needed to, persuade a public that then fears, or is ignorant of, the facts. In a similar way, other political events have been evaluated in the context of a manifested clash between proponents of liberal cosmopolitanism and defenders of socially conservative values (Norris and Inglehart 2019). The March for Science movement – a global manifestation in support of science and scientists – underpinned this opposition through arguments, narratives, and images it used to oppose a fact-oriented ‘elite’ and an emotional, ignorant ‘people’ (Durnová 2019a).

## Conclusion

The time has come to put to rest to the calling out of who is responsible for the post-truth mess. This is probably the most fundamental message of Fischer’s analysis. We should ask what social constructivism can do for us to fix the mess, instead of reiterating the discussion of who is the villain. In this brief commentary, I, therefore, suggested to complement Fischer’s analysis of science denial with discourses on emotions that show how reference to emotions make specific positions illegitimate and opposed to facts.

After all, these questions surrounding the production of evidence to inform policy debates are not limited to critical policy studies. On the side of mainstream approaches, there have been important debates on how analysts should deal with the fragility of knowledge and related ignorance when trying to influence policy with their analytical insights (Ayres 2014). Even more importantly, debates on how policy scholars can steer a better discourse among policy theories through a reflective use of the vocabulary to describe concepts and analytical tools gained prominence (Weible and Cairney 2018). Other traditions in policy studies could, and should, join critical policy studies in pinpointing their added value in addressing the post-truth phenomenon. Proposing conceptual ways to bring a diversified and subtle notion of emotions as a legitimate part of policy debates, on science and even beyond it, could be the joint aim to appease the appeal of post-truth.
